# Efficient Formation of Size-Selected Clusters upon Pickup of Dopants into Multiply Charged Helium Droplets

**DOI:** 10.3390/ijms23073613

**Published:** 2022-03-25

**Authors:** Siegfried Kollotzek, Olga V. Lushchikova, Lukas Tiefenthaler, Fabio Zappa, Paul Scheier

**Affiliations:** Institut für Ionenphysik und Angewandte Physik, Universität Innsbruck, Technikerstr. 25, A-6020 Innsbruck, Austria; siegfried.kollotzek@uibk.ac.at (S.K.); lukas.tiefenthaler@gmail.com (L.T.); fabio.zappa@uibk.ac.at (F.Z.); paul.scheier@uibk.ac.at (P.S.)

**Keywords:** size-selected cluster, helium droplets, mass spectrometry

## Abstract

Properties of clusters often depend critically on the exact number of atomic or molecular building blocks, however, most methods of cluster formation lead to a broad, size distribution and cluster intensity anomalies that are often designated as magic numbers. Here we present a novel approach of breeding size-selected clusters via pickup of dopants into multiply charged helium nanodroplets. The size and charge state of the initially undoped droplets and the vapor pressure of the dopant in the pickup region, determines the size of the dopant cluster ions that are extracted from the host droplets, via evaporation of the helium matrix in a collision cell filled with room temperature helium or via surface collisions. Size distributions of the selected dopant cluster ions are determined utilizing a high-resolution time of flight mass spectrometer. The comparison of the experimental data, with simulations taking into consideration the pickup probability into a shrinking He droplet due to evaporation during the pickup process, provides a simple explanation for the emergence of size distributions that are narrower than Poisson.

## 1. Introduction

Clusters and nanoparticles have sometimes remarkably different properties than the corresponding bulk materials. This is due to the enhanced surface to volume ratio which is proportional to 1/*r* and for very small particles due to quantum confinement [1], electronic [2] and geometric [3] shell closings or super atomic character [4]. For small clusters, the addition or removal of a single atom can have a substantial effect on its electronic [5], optical [6] and magnetic [7,8] properties as well as on its stability [9] and chemical reactivity [10,11]. Clusters can be made by either top-down [12] (breakup of larger particles or bulk) or bottom-up [13] (condensation of atoms or molecules) approaches. In the case of metal clusters, wet chemical synthesis has been successfully utilized to form specific size-selected clusters but most of the time organic ligands are required to prevent further agglomeration [14]. The influence of these ligands on the electronic character of the cluster may have negative effects and this wet chemical approach is limited to only specific cluster sizes and metals. In contrast, gas phase approaches such as supersonic expansion or gas aggregation enable the formation of clusters of every material and size. However, these methods lead to the formation of a broad distribution of cluster sizes which typically has a log-normal shape, often superimposed by intensity anomalies that reflect the stability of the individual cluster sizes [2,3]. In the case of several metal clusters, spin pairing leads to a pronounced odd–even oscillation of the ion yield as a function of the cluster size [2,15]. For metals with an unpaired s-orbital electron, such as the alkali or coinage metals, closed shell singly charged ions turn out to switch into weakly bound open shell electronic systems upon neutralization. Selection of a single cluster size is often achieved by mass spectrometry of ionized clusters. With increasing cluster size and cluster sizes that exhibit low abundance in the ionic cluster size distributions, such as M_10_^+^ (M = Na, K, Ag or Au) this process discards often >95% of the initially formed cluster material.

In the present paper, we present a novel approach to obtain intense beams of mono-dispersed singly charged cluster ions of almost any kind via pickup of gas phase dopants into multiply charged helium droplets. Droplets containing up to several billion helium atoms [16,17,18] can be formed via expansion of precooled pressurized helium through small nozzles into an ultra-high vacuum [19,20]. Evaporative cooling leads to an isothermal temperature of these droplets of about 0.4 K [21] and collisions with dopants lead to pickup and aggregation of these species to clusters [22,23] and nanoparticles [24] inside (heliophilic dopants) or on dimples at the surface of the helium droplets (heliophobic dopants) [25,26]. A convolution of the size distribution of the helium droplets and the Poisson pickup statistics lead to a broad log-normal size distribution of the dopant clusters. Both transmission electron microscopy images of deposited nanoparticles grown in neutral helium droplets [24,27] and mass spectrometry of dopant cluster ions ejected from the host droplets upon electron ionization clearly demonstrate this fact [20]. Furthermore, all mass spectra measured by this method reveal the same intensity anomalies (often designated as magic numbers) [2,3] that are observed via formation of the same cluster ions devoid of helium droplets [20].

Recently, Laimer et al., discovered that helium droplets containing 38 million He atoms can accommodate up to 55 charges [28]. Tiefenthaler et al. [29] reversed the order of pickup and ionization and demonstrated that pickup into multiply charged helium nanodroplets has several advantages concerning the yield and size distribution of dopant cluster ions. Each of the charge centers in a multiply charged helium droplet acts as a seed for the growth of a singly charged dopant cluster, as neutral dopants are polarized and pulled to the nearest charge. Instead of only one neutral dopant cluster upon pickup into neutral helium droplets, many singly charged dopant clusters are formed simultaneously in multiply charged helium droplets. This explains the high yield of dopant cluster ions formed by this method. Tiefenthaler et al. extracted charged dopant clusters from the host droplets by shrinking the droplets via collisions with room temperature He gas. Coulomb repulsion ejects charged dopant clusters as soon as the size of the droplets drops below a critical size [28]. The cluster size distributions obtained by this method often exhibit no magic numbers and their shape is narrower compared to experiments where doped neutral helium droplets are ionized [20]. However, we did not have an explanation for these unusually narrow size distributions at that time.

In this paper, we elaborate further on the method described in detail in Tiefenthaler et al. [29] and show that the application of multiply charged helium nanodroplets is not only useful to tune the average cluster size, control the number of attached He-atoms and produce intense cluster beams, but it can also significantly narrow down the cluster size distribution. A pickup mechanism is proposed to explain how the obtained cluster size distributions of different dopants (Ag, Au, Na and C_60_) change from log-normal to nearly Poisson distributions, and, under special conditions, sometimes even become narrower than a Poisson distribution with the same expectation value. The possibility of producing such narrow distributions opens new avenues since the desired cluster sizes can be directly produced in a high amount without additional mass selection, leading to the loss of the major fraction of dopant material.

## 2. Results

### 2.1. Cationic Silver Clusters

**Figure 1:**
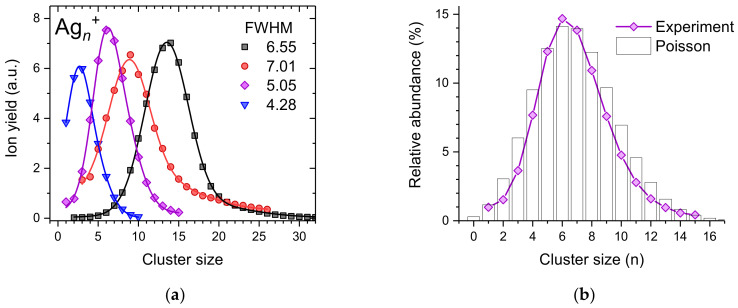
(**a**) Measured size distributions of cationic silver clusters formed upon pickup into charged helium droplets (symbols). The lines are fits to the data using a superposition of two Gaussian peak functions; (**b**) Comparison of one measured cluster size distribution with a Poisson distribution having an expectation value of λ = 7.9.

Figure 1a shows a few selected cluster size distributions of silver cluster ions resulting from the pickup of atomic silver vapor (formed via evaporation of bulk silver in an ohmically heated oven) into mass-per-charge selected helium droplets (~2 × 10^5^ He atoms per charge at an average charge state of $\bar{z}$ = 10). Depending on the number of He atoms per charge and the silver vapor pressure, it is possible to shift the average cluster size to any desired value. In the present case, we changed the pressure of the silver vapor by setting the temperature of the oven between 994 K and 1046 K. With increasing temperature, the average silver cluster size increases. All four experimentally determined cluster size distributions (solid symbols) exhibit relatively narrow asymmetric peak shapes that can be effectively reproduced by a superposition of two Gaussian peak functions (solid lines). In the case of small average cluster sizes (blue triangles and magenta diamonds), the centers of the two Gaussians are less than 1.3 cluster sizes apart, whereas this difference increases to up to 5.2 for larger average cluster sizes.

The binding energy of a silver atom to a cationic silver cluster was determined via density functional theory [30] and a model-free method that uses a combination of sequential and single step decays [31] to range from 1.57 eV to 2.74 eV, depending on the cluster size. This binding energy is quickly dissipated into the He matrix and leads to the evaporation of up to 4560 He atoms. Since we selected droplets that contain about 2 × 10^5^ He atoms per charge, even after quenching the binding energy of an Ag_10_^+^ cluster, 1.5 × 10^5^ He atoms per charge remain in the droplets. However, the He atoms that are lost from the droplets collide with the wall of the oven and can only escape through openings with a diameter of 2 mm at both sides of the oven. Thus, hot helium gas will also contribute to the evaporation of the droplets. Whenever the size of a droplet drops below its critical size for a given charge state, a charged silver cluster is ejected together with a small solvation layer of He around it. Under extreme pickup conditions (black squares and red circles), the liberated silver cluster ions will be deprived from all attached He atoms and warmed up by the He gas to a point where they start to evaporate silver atoms. Ejected silver cluster ions and the remaining He droplets proceed to a radio frequency hexapole ion guide, filled with room temperature helium gas [29]. Collisions with He atoms shrink the droplets, liberate additional silver cluster ions and remove the He potentially attached to them. In the case of strongly bound metal clusters, such as silver or gold, the energy input by collisions of He at room temperature is not sufficient to cause the loss of metal atoms. In the case, where only a small amount of silver was picked up, a substantially larger helium droplet has to be vaporized, which requires a higher He gas pressure in the evaporation cell (for the blue triangles a pressure of 5.2 Pa was used and for the black squares only 1.1 Pa was sufficient).

Only the cluster size distribution designated with red circles exhibits a weak magic number at *n* = 9, indicated by this data point clearly outside the corresponding fit to the cluster size distribution. For this measurement, the oven was operated at its highest temperature. Therefore, silver cluster ions ejected from the host droplet are quickly deprived from their remaining He solvation layer and prone to collision-induced fragmentation inside the 1046 K hot pickup cell leading to the emergence of magic numbers. Figure 1b shows a comparison of the cluster size distribution designated with magenta diamonds in Figure 1a and a Poisson distribution with an expectation value of λ = 7.9 (black bars). It is interesting to note that the measured cluster size distribution is narrower than this Poisson distribution which could be expected for pickup into size-selected helium droplets with a size that does not change due to evaporation of helium atoms during the pickup process.

**Figure 2:**
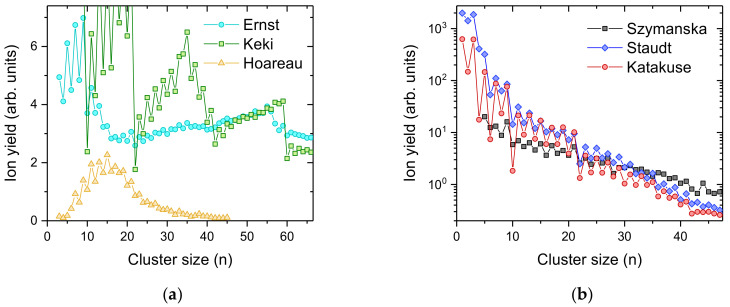
Cluster size distributions of positively charged silver clusters Ag_n_^+^ taken from the literature and made by different methods. In every case, the mass spectra exhibit pronounced intensity anomalies that can be assigned to spin pairing of the 5 s electrons and electronic shell closures most noticeably at *n* = 9, 21, 41 and 59. (**a**) shows silver cluster size distributions obtained by Ernst and Hauser (cyan circles, electron ionization of neutral Ag doped helium droplets [49]), Keki et al. (green squares, MALDI [47]) and Hoareau et al. (yellow triangles, inert gas condensation [41]); The cluster size distributions in (**b**) show an almost exponentially decreasing intensity with increasing cluster size from Szymanska et al. (black squares, laser desorption/ionization of silver benzoate [48]), Staudt et al. (blue diamonds, 15 keV Xe^+^ ion sputtering [46]) and Katakuse et al. (red circles, 10 keV Xe^+^ ion sputtering [15]).

Figure 2 shows several cationic silver cluster size distributions found in the literature. Clusters and nanoparticles of coinage metals have been extensively studied in the past [5,32] due to their potential as catalysts [33,34,35], and their optical [36,37] and biomedical applications [38]. In particular, silver turns out to have pronounced antimicrobial properties [39,40]. Silver clusters have been formed via various techniques, such as inert gas condensation [41]; direct laser vaporization [42]; laser vaporization into a buffer gas and supersonic expansion [43]; ion sputtering [44,45,46]; MALDI [47,48] and pickup into helium droplets [49]. Common to all these studies are pronounced intensity anomalies in the cluster size distributions determined by mass spectrometry (see Figure 2). Electronic shell closures at *n* = 9 and *n* = 21, corresponding to 8 and 20 unpaired 5 s electrons, superimposed onto an odd–even oscillation due to spin pairing are clearly observed, irrespective of the average size of the silver cluster ion distributions. In contrast to the present experimental data shown in Figure 1, most of the data taken from the literature (Figure 2) were measured most likely with silver having a natural isotopic composition (^107^Ag:^109^Ag = 0.514:0.486). This distributes the ion yield among different masses and reduces the height of the most abundant peak with increasing cluster size. All cluster size distributions, except for the experiments by Ernst and Hauser [49] and Szymanska et al. [48], were determined from the peak heights of the published mass spectra. It is not clear how the ion yield was determined in the other two studies, i.e., peak heights (most abundant isotopologue) or peak areas (sum of all different isotopologues). A correction from areas to peak heights in these cases leads to a decrease in the ion yield with increasing cluster size and better agreement with the other data.

In Table 1 we compare the relative amount of silver that is ending up in the cluster size *n* = 10 and the one with the highest yield in each cluster size distribution shown in Figure 1 and Figure 2. For silver, with its natural isotopic distribution, the peak height of the isotopologue with the highest abundance decreases with increasing cluster size. The correction for isotopic distribution only affects the most abundant silver cluster from the measurements of Staudt et al. [50] and Katakuse et al. [47], by shifting it from *n* = 1 to *n* = 3. Please note that the cluster size distributions published by Keki et al. [47] and Szymanski et al. [48] are incomplete with missing data points for several of the most likely highly abundant cluster sizes. Only the very early measurement by Hoareau et al. [41] and the recently published cluster size distributions by Ernst and Hauser [49] exhibit a distribution that peaks at an *n*, max > 1. Since Ag_10_^+^ is the cluster size following an electronic shell closure at *n* = 9, its intensity in experiments that are prone to dissociation reactions of hot cluster ions is very low. Utilizing the presently described charged droplet pickup technique, it is possible to find more than 10% of all silver atoms of the cluster size distribution in Ag_10_^+^, by tuning the pickup pressure to the right value. In contrast, all previous experiments achieved between a factor 5 to 50 less intensity in this cluster size. When looking at the most intense cluster ions formed in each experiment, the charged droplet pickup technique is at least a factor two more efficient, particularly when ignoring monomers.

### 2.2. Cationic Gold Clusters

**Figure 3:**
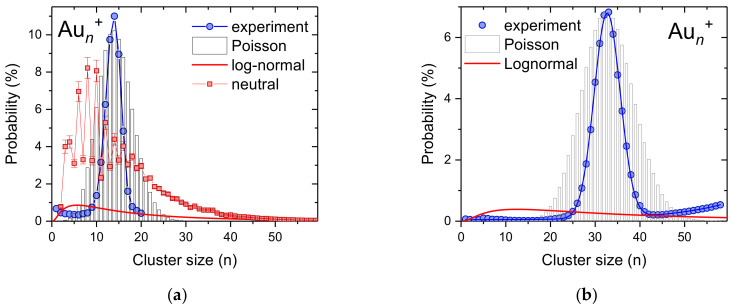
Measured size distribution of gold cluster ions obtained via pickup of gold atoms into highly-charged helium droplets (blue circles, 6 × 10^4^ He atoms per charge). At an oven power of 140 W the average size of the Au*_n_*^+^ clusters is 13 (**a**) and for 190 W the gold clusters contain an average of 33 gold atoms (**b**). The bar graph are Poisson distributions with expectation values corresponding to the average cluster sizes and the red lines are log-normal distributions. The red squares in (**a**) represent a Au*_n_*^+^ cluster size distribution obtained via electron ionization of neutral He droplets doped with gold. The average cluster size of this distributions is also 13.

For gold, which has only one stable isotope, we also performed similar measurements, as in the case of silver clusters. Figure 3 shows two cluster size distributions obtained via pickup of gold vapor into multiply charged helium droplets that were formed as neutrals with an average size of about 2 × 10^6^ He atoms and were charged via electron impact at 40 eV electron energy and 500 µA electron current (blue points). With the electrostatic quadrupole bender, we selected droplets that contain between 2.5 × 10^5^ and 4.8 × 10^5^ He atoms per charge. At a heating power of 140 W, the resulting gold cluster size distribution peaks at *n* = 13 (Figure 3a), while by increasing the oven power to 190 W the most abundant gold cluster shifts to *n* = 33 (Figure 3b). In both diagrams, we plotted a Poisson distribution (bar graphs) to which we normalized the experimental data. In both cases, the measured cluster size distribution is substantially narrower than the corresponding Poisson distribution. This is surprising, since for a monodisperse helium droplet where the mass loss due to evaporative cooling during the pickup process can be neglected, one can expect a pure Poisson cluster size distribution [50].

### 2.3. Cationic Sodium Cluster Ions

To verify whether the use of charged He droplets can also give more control over the size distribution and the presence of magic numbers of heliophobic dopants such as alkali metals [51], the formation of cationic sodium clusters upon pickup into multiply charged helium droplets has been studied. In contrast to heliophilic silver and gold, where every atomic dopant submerges into the He droplet, neutral sodium atoms reside in dimples on the surface of helium droplets due to the interplay between attractive polarization forces and short-range Pauli repulsion. Stark and Kresin [52] calculated a critical size of *n* = 20 for submersion of neutral sodium clusters into helium which was later experimentally confirmed by An der Lan et al. [53]. Later, several experimental [54] and computational [25,55,56] papers were studying the submersion of a heliophobic dopant by the presence of a heliophilic dopant inside a helium droplet. The stronger interaction between a charge center and neutral dopant compared to two neutral dopants as well as the location of charge centers close to the surface in the case of multiply charged helium droplets should lead to an efficient submersion of sodium atoms and sodium cluster ion formation.

**Figure 4:**
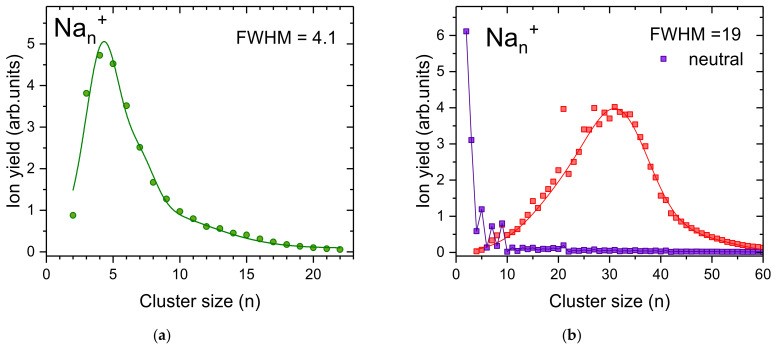
Pickup of sodium into charged helium droplets leads to relatively narrow cluster size distributions. At low sodium vapor in the pickup cell (**a**) no magic number are observed whereas high sodium vapor pressure (**b**) leads to larger Na_n_^+^ clusters and the emergence of magic numbers at the well-known electronic shell closures *n* = 9 and *n* = 21, as well as an odd-even oscillation of the ion yields at small cluster sizes. The distributions are fitted with multiple Gaussians and FWHM is determined. In (**b**) we also show a sodium cluster size distribution obtained via electron ionization of neutral He droplets doped with sodium (purple squares, data taken from An der Lan et al. [53].

Two types of distributions could be achieved by varying the droplet size and evaporation pressure (Figure 4). Sodium cluster ions containing up to *n* = 23 atoms and peaking at *n* = 4 are formed in relatively small He droplets with 4.6 × 10^5^ He atoms per charge at a maximum oven temperature of 403 K (Figure 4a). Increasing the He droplet size to 5.6 × 10^5^ atoms per charge leads to a much broader cluster size distribution as illustrated in Figure 4b (red squares), with Na_n_^+^ cluster ions up to *n* = 60. The formation of these larger sodium cluster ions requires a higher temperature in the oven (up to 594 K). As in the case of silver cluster ions discussed above, the high oven temperature leads to massive pickup of sodium atoms. Therefore, the energy of the sodium cluster ion formation results in a substantial He evaporation. After collisions with the oven surface the hot helium gas contributes to the evaporation of helium from the droplet and ejection of the charge center. This finally leads to the evaporation of sodium atoms from ejected charge centers that were deprived from their helium solvation layer. The large difference in the binding energies of small sodium cluster ions due to spin pairing and electronic shell closures is a prerequisite for the emergence of magic numbers upon cluster fragmentation. Since a large fraction of He is already evaporated from the droplet in the pickup oven, much less He pressure is required in the evaporation cell to liberate the remaining Na cluster ions embedded in the helium droplet. A total of 0.2 Pa of room temperature He is enough for high-temperature oven measurements (Figure 4b), while 0.73 Pa is required for a low oven temperature (Figure 4a).

A comparison of the presently obtained cluster size distributions with data obtained via electron ionization of helium droplets doped with sodium (Figure 4b, purple circles) clearly shows the potential of pickup into multiply charged helium droplets to form clusters of a desired size in high abundance. Nevertheless, the oven temperature is quite crucial, since too high temperatures can lead to unwanted Na evaporation, and as a result to anomalies in the cluster size distribution by magic numbers.

### 2.4. Positively and Negatively Charged Fullerene Clusters

**Figure 5:**
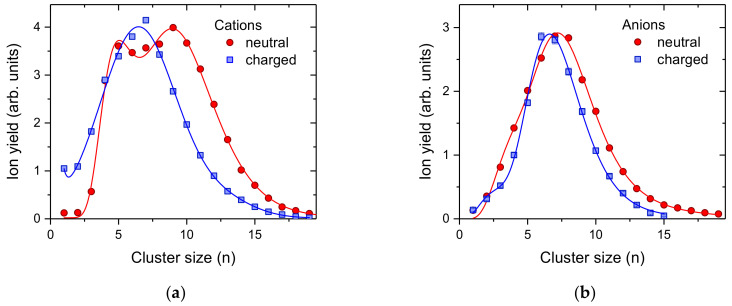
Cluster size distributions of positively (**a**) and negatively (**b**) charged of C_60_ clusters formed upon pickup into pre-charged helium droplets (blue squares) and electron bombardment of neutral He droplets doped with C_60_. In the case of cations, the electron energy was set to 50 eV and the mass spectrum for anions was set to 22 eV, at which resonant formation of He*^−^ [61] is essential for the production of anionic dopant (clusters).

As a final example we investigated the formation of C_60_ cluster ions of both polarities via pickup into pre-charged helium droplets. We performed these measurements with two different configurations of the same instrument, i.e., electron ionization of neutral He droplets doped with C_60_ (red circles, Figure 5) [57] and surface impact of charged helium droplets subsequently doped with C_60_ (blue squares, Figure 5) [58]. The average size of the He droplets initially formed were in all cases about 10^6^ He atoms [59]. He droplets of this size can easily accommodate more than 10 positive charges, however only one anionic charge center [60]. The critical size for negatively charged He droplets carrying two charges is 4 million He atoms. However, in a log-normal distribution with an average value of 4 million, about 8.2% of the droplets contain more than 4 million He atoms that could potentially carry two charges (the probability for triply charging of droplets larger than 15.7 million He atoms is less than 0.3%). Pickup into such large neutral He droplets results in large fullerene clusters due to the high geometric (capturing) cross section of these droplets. If more than one charge center is present, the fullerenes that are picked up will be shared among the charge centers, which results in more than one smaller fullerene cluster ions. Since negatively charged droplets have huge critical sizes for multiply charging [60], under the present conditions, the helium droplets are most likely singly charged. This agrees well with the small difference between pickup into neutral (red circles in Figure 5b) and negatively charged (blue squares in Figure 5b) droplets. In contrast, the yield of large C_60_^+^ clusters in the case of pickup into charged He droplets is reduced compared to pickup into neutral He droplets and positive ionization. For cations (Figure 5a), the presence of multiply charged He droplets is much more likely with charge states of up to 50. In order to provide enough C_60_ for each charge center, the temperature of the oven vaporizing the fullerene powder was increased from 503 K to 549 K. Again, pickup into charged He droplets, even without mass-per-charge selection, results in a narrower size distribution of the dopant cluster ions formed.

## 3. Discussion

All three examples presented in this work clearly show that pickup of dopants into preferentially highly-charged helium droplets leads to intense formation of singly-charged dopant clusters with exceptionally narrow cluster size distributions. In several cases, the measured size distribution was narrower than a Poisson distribution with the same average value, as shown in Figure 1b and Figure 3. In order to shed light on the mechanisms that define the resulting size distribution of charged dopant clusters, we developed a simple model that simulated the pickup process for mass-per-charge selected helium droplets based on the presently chosen experimental parameters (number of helium atoms per charge, average size of the initially formed helium droplets, density of dopants in the pickup cell), the binding energy of dopants to a singly charged cluster of the same material (about 2.5 eV for the coinage metals silver [30] and gold [62] and 0.7 eV for sodium [63]) and the critical sizes for multiply charged helium droplets (about 5 × 10^4^ He atoms per charge for the charge states below *z* = 10 [28]).

**Figure 6:**
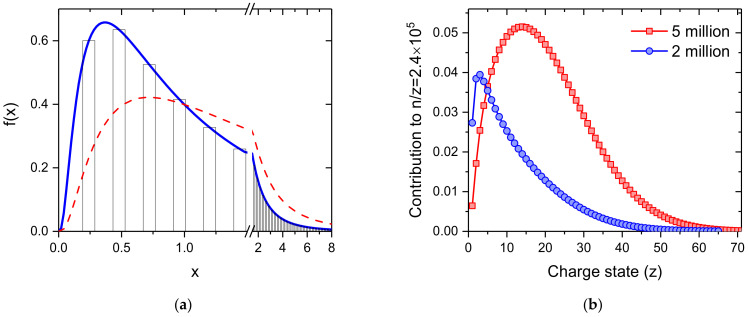
(**a**) log-normal distribution with an average value of 1, resembling the size distribution of the neutral helium droplets. An electrostatic quadrupole bender selects differently charged droplets having the same mass-per-charge value, indicated by the bars. The red dashed line indicates the amounts of dopants being picked up by the differently sized He droplets; (**b**) shows the relative ion yield of different charge states the mass-per-charge selected helium droplets with *n*/*z* = 2.4 × 10^5^ He atoms per charge for neutral log-normal droplet size distributions having average sizes of 2 million (blue circles) and 5 million (red squares) He atoms.

As a first step, a log-normal distribution (see Figure 6a)

$$f\left(x\right)=\frac{1}{\sqrt{2\pi }x}exp\left(-\frac{ln{\left(x\right)}^{2}}{2}\right)$$

with

(1) $$x=\frac{n}{\bar{n}}$$

is considered for the neutral helium droplets. The size of a helium droplet divided by the average size of the distribution is calculated to determine the intensity of all helium droplets with the same mass-per-charge value. Due to the shape of the log-normal distribution we take up to eight times the average value of the neutral droplets into consideration, since the intensity at this size drops below 1% of the maximum of the distribution which is found at *x* = 0.3679. The cross section for pickup of dopants is proportional to the surface size of a droplet and thus to *n*^⅔^. The red dashed line shows the amounts of dopants being picked up by He droplets as a function of their size. The electrostatic quadrupole bender selects charged helium droplets having a specific mass-per-charge value. The charge distribution of helium droplets of a specific size *n* is strictly Poisson distributed with an expectation value determined by the geometric cross section of the droplet, its speed, the electron current and the size of the cross section of the electron beam. The contribution of the droplet size to a specific mass per charge value *n*/*z* is obtained by multiplication of the yield of a droplet size *n*, according to the log-normal distribution, and the probability of charge state *z* for that *n*/*z*. Figure 6b shows these contributions for a mass-per-charge value of 2.4 × 10^5^ and neutral droplet size distributions with average sizes of 2 and 5 million He atoms, indicated by the blue circles and red squares, respectively. This figure clearly indicates the importance to include droplets much larger than the average size (here at about *z* = 4 and 21) into the simulation.

To obtain a reasonably small statistical uncertainty, about 50,000 droplets are simulated and a realistic spread of ±5% for the size is randomly added to each droplet (resolving power of the quadrupole bender). The size (*n*_0_ = number of helium atoms) of a droplet determines its pickup cross section σ via its surface which is proportional to *n*_0_^⅔^. All charge centers in a multiply charged droplet have the same probability to capture a dopant that was picked up. The binding energy BE of a dopant to a charged dopant cluster is released to the surrounding He matrix and each eV dissipated leads to the evaporation of 1600 He atoms. This reduction of the He droplet size lowers the cross section for further pickup processes according to:

σ1=σn0−1600·i·BEn023

If the size of a multiply charged droplet drops below the critical value for keeping all its charges, the charge center with the lowest binding energy to the droplet is ejected. Since the largest dopant cluster released most binding energy, we expect that this dopant cluster is most likely removed and thus stops growing. The other remaining charge center continue to attach dopants until the droplet size shrinks again below the critical value for this charge state. With decreasing number of charge centers *z*_c_, more He per charge center is available to be vaporized, i.e., 5 × 10^4^/*z*_c_, which becomes highest for the last remaining charge, i.e., *z*_c_ = 1.

**Figure 7:**
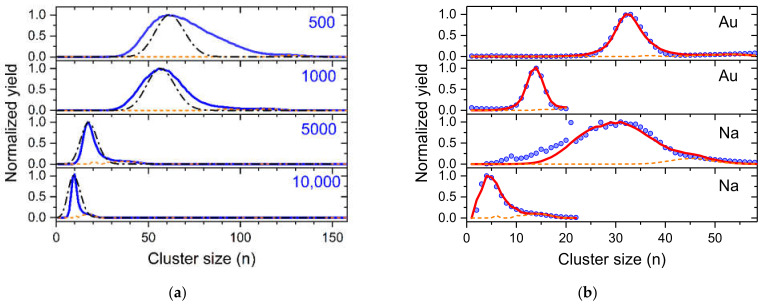
(**a**) Demonstration of the importance of the number of helium atoms evaporated in each doping event to the resulting cluster size distribution (see text). The values in the diagrams indicate the number of He atoms that are evaporated per dopant being picked up. The dash-dotted black lines are Poisson distributions with expectation values of the position of the maximum of the corresponding simulated cluster size distribution; (**b**) Comparison of experimental cluster size distributions (symbols) shown above and simulated cluster size distributions (red solid lines). The dashed orange line in both (**a**,**b**) indicates the last dopant cluster remaining in the droplet which under heavy doping conditions grows to considerably larger sizes.

With a python program, shown in the Supplementary Materials, we simulate various cluster size distributions. Figure 7a shows simulated dopant cluster size distributions for pickup of 2000 potential dopants (each attempt has a probability for capture based on the current cross section of the droplet) into charged helium droplets with *n*/*z* = (2 ± 0.1) × 10^5^ and neutral droplets with an average size of 10^6^ He atoms. Each pickup process leads to the evaporation of 500 up to 10,000 He atoms, as indicated in the four diagrams. The evaporation of 500 He atoms per successful pickup event is barely enough to evaporate the complete droplet to a stage where only one charge center remains (orange dashed lines). The resulting cluster size distribution has a shape similar to a log-normal distribution (upper panel) and is clearly wider than a Poisson distribution (dash-dotted black line). However, massive pickup and evaporation of He to a point where most charge centers are ejected inside the pickup cell results in narrow cluster size distributions often followed by a pronounced satellite peak due to the extra growth of the last remaining charge center (orange line). The width of the resulting cluster size distribution is narrower than a Poisson distribution (black dash-dotted line in the lower diagram).

In Figure 7b we compare four experimentally determined cluster size distributions for gold (Figure 3) and sodium (Figure 4), designated with symbols with simulated distributions by optimizing the parameters (average neutral size, *n*/*z*, evaporation per pickup, number density of dopants). The values of these parameters are listed in Table S1 in the Supplementary Materials. In three cases we achieved perfect agreement to the experiments and the resulting parameters were very reasonable in comparison to the experimental conditions. Only the sodium cluster size distribution that was obtained at the highest oven temperature (third diagram from above) cannot be simulated. The presence of clear magic numbers in the experimental data at *n* = 9 and 21 indicates fragmentation of the sodium clusters in the hot pickup cell. Such processes lead to the formation of smaller cluster ions which is not included in our model. For high vapor density of dopants, the amount of He that is evaporated from the droplets will contribute to additional shrinking of the droplets as the fast He atoms reflection from the oven walls will also collide with the droplets. Currently, this is only included in the number of He atoms evaporated per pickup event. In the case of the narrow sodium and gold distributions, about twice the amount of He per pickup event has to be evaporated to obtain these results.

According to our simulations, the most efficient way to produce narrow dopant cluster size distributions is to use a high pickup pressure where most charge centers are ejected inside the pickup cell. The distance between charge centers at the critical size for Coulomb ejection is about 30 nm. This results in a kinetic energy release due to the Coulomb repulsion of the remaining charged droplet in the order of 50 meV, with most of it going into the ejected dopant cluster ion. This energy is negligible in comparison to the kinetic energy of Au_10_^+^ of 410 meV due to the velocity of the droplet (typically 200 m/s [59]). However, in the case of light dopants, such as sodium, this kinetic energy release becomes comparable to the initial forward energy. Therefore, we expect the loss of low-mass dopant clusters if ejected sideways from the path of the droplets. This will lead to a suppression of the low-mass side of the measured cluster size distributions.

In their famous review article “Catalysis by clusters with precise numbers of atoms” Tyo and Vajda write, “The limiting factor in all cluster studies is creating a sufficiently high concentration of the desired species and separating them from the overall distribution formed during cluster generation.” [10]. This statement has been confirmed in numerous cluster experiments. In the present study, we demonstrate the possibility of forming singly charged clusters via pickup of various dopants into multiply charged helium droplets with cluster size distributions that are much narrower compared to conventional methods, typically leading to log-normal distributions. Based on the simulation of the pickup process, we could identify crucial parameters to achieve size distributions that are beating Poisson distributions with the same expectation value. This knowledge will help in future experiments to produce any desired cluster size with highest efficiency. In this way, the costs for expensive dopant materials, such as platinum or palladium, can be significantly reduced since most dopant material will be used to create clusters of a specific desired size, rather than clusters that spread over a wide range of sizes. Moreover, the high intensity of the particular cluster size of interest is very favorable for subsequent spectroscopic studies or cluster deposition, as it reduces the time for the data acquisition or deposition. In addition, all other features that were demonstrated for pickup into neutral helium droplets, such as sequential pickup, formation of core-shell clusters, formation of He tagged ions are still viable. Some preliminary experiments indicate that this method can also be utilized for the formation of almost mono-dispersed metal nanoparticles consisting of several thousand atoms. Currently we are designing a new experimental setup to deposit such large nanoparticles onto amorphous carbon films and analyze them in a transmission electron microscope, similar to the previous studies in the groups of Ellis [24] and Ernst [13,64,65].

## 4. Materials and Methods

Helium droplets were formed via expansion of pressurized and helium of highest grade (6.0, Messer Austria GmbH, Vomp, Austria) through a pinhole nozzle (5 μm nominal diameter, A0200P, Günther Frey GmbH & Co. KG, Berlin, Germany, 5.67 μm exact diameter determined by the scanning electron microscopy) into a vacuum chamber with a base pressure below 1 µPa. The helium droplet source was attached to close-cycle cryocooler (RDK-408D2, Sumitomo Heavy Industries, Ltd., Darmstadt, Germany). The temperature of the helium before expansion was measured with a silicon diode (Lakeshore DT-670 in CU package) attached closely to the nozzle and used as an input for a PID regulator (Lakeshore Temperature Controller Model 335) that controlled the current passing through a resistor attached to the droplet source. This enabled source temperatures between 4.2 K and 25 K with ±0.1 K precision. For the present study source temperatures were used between 7 K and 9.7 K. At a stagnation pressure of 2 MPa, this resulted in average droplet sizes between 20 and 1 million He atoms [59]. After passing a 0.5 mm skimmer (Beam Dynamics, Inc., Dallas, TX, USA) positioned about 5 mm from the nozzle, the helium droplets were ionized via electron bombardment. The electron energy was set between 40 eV and 70 eV and electron currents were used between 150 µA and 550 µA. At these conditions the helium droplets were multiply charged to the highest possible value which is 12 for droplets containing 1 million He atoms and 100 for droplets containing 20 million He atoms [28]. Two instruments were utilized in the present study. In the first one, the charged droplets passed an electrostatic quadrupole bender that selected droplets of a specific mass-per-charge value to enter differentially pumped pickup chambers where they were doped with atomic or molecular species. Solid materials, such as monoisotopic silver (99.67% ^109^Ag, STB Isotope Germany GmbH, Hamburg, Germany), gold (99.9%, Ögussa GmbH, Innsbruck, Austria), sodium (99.95%, Sigma Aldrich, Vienna, Austria) or C_60_ (99.99%, SES Research Inc., Houston, TX, USA) were vaporized in ohmically heated ovens. More volatile dopants can be introduced via a heated gas inlet often controlled by a regulated leak valve or flowmeter. The charged helium droplets picked up monomeric dopant species on their fly through the pickup cell. The cross section for capturing a dopant depends primarily on the geometric cross section of the helium droplet. Ion-induced dipole interaction between the charge centers in the helium droplet pulled the dopants to the nearest charge center. Charge transfer from the He_n_^+^ snowball to the first arriving dopant ionized the latter in an often highly exothermic reaction, resulting from the difference in the ionization energies of the dopant and He. In the case of fragile molecules, dissociation may occur. Recently, Albertini et al. demonstrated that the neutral fragments formed via a dissociative ionization process inside a He droplet can be captured by the ionic fragment via ion-induced dipole interaction and energy dissipation into the surrounding helium matrix [66]. Furthermore, pre-doping the charged helium droplets with a small amount of hydrogen, leading to proton transfer ionization from (H_2_)_*n*_H_3_^+^ (*n* ≥ 0) to almost all dopants except O_2_, Ne and Ar [67] leads to the soft chemical ionization of dopants free of fragmentation.

In the present study, we utilized two different methods to extract singly charged dopant cluster ions from the massive helium droplets: (i) Multiple collisions with room temperature helium gas in an ion guide (RF-hexapole) shrinks the droplets and leads to a sequential ejection of charged dopant clusters whenever the Coulomb repulsion exceeds the binding energy of these charges to the droplet [29]. Depending on the helium pressure in the collision cell, the dopant cluster ions are deprived of helium or still solvated with up to a few hundred helium atoms and (ii) Most of the charge centers of ionized helium droplets are liberated from the droplet and backscattered upon surface impact, as recently demonstrated by Martini et al. [58]. All low-mass ions are then guided to the extraction region of an orthogonal time of flight mass spectrometer and cluster size distributions are obtained from high-resolution mass spectra (m/Δm up to 15,000).

## Figures and Tables

**Table 1:** Relative amount (in %) of silver in Ag_10_^+^ and the most abundant silver cluster ion formed in the experiments shown in Figure 1 and Figure 2. Italic fonts indicate that these values are upper limits since some mass peaks were off-scale in the respective studies.

Data Sets Shown in Figure 1 and Figure 2 (Literature)	Ag_10_^+^	*n*, max	Ag_*n*,max_^+^
Figure 1, blue triangles	0.62	3	20.62
Figure 1, magenta diamonds	8.34	6	15.41
Figure 1, red circles	10.8	9	11.09
Figure 1, black squares	4.31	14	13.32
Ernst	0.48	9	0.82
Keki	<0.23	-	-
Hoareau	1.89	15	5.99
Szymanska	*2.07*	*5*	*3.55*
Staudt	0.72	1	9.94
Katakuse	0.23	1	7.76

## Data Availability

All data are in electronic form available via a data server hosted by the University Innsbruck.

## Supplementary Materials

The following supporting information can be downloaded at: https://www.mdpi.com/article/10.3390/ijms23073613/s1.
