# Supplementary material for: Efficient Formation of Size-Selected Clusters upon Pickup of Dopants into Multiply Charged Helium Droplets
Source: Int J Mol Sci. 2022 Mar 25;23(7):3613. doi: 10.3390/ijms23073613 (PMC8998201; doi:10.3390/ijms23073613)
Supplement: Supplementary file 1 [file ijms-23-03613-s001.zip › File S1-Python code to simulate the cluster size distributions.pdf]

```
import numpy as np
import matplotlib.pyplot as plt
import math as m
import random

def Poisson(Charge,Lamda):
   prob = m.exp(-Lamda)
   for i in range(1, Charge):
      prob = prob * Lamda / i
   return(prob)

def CalcDist(AverageNeutral, SizePerCharge, evap, runs, PU_Dens, HeLoss, sigma0,
   PlotGraph):
   count=0
   fields=int(8.0*AverageNeutral/SizePerCharge)                    # max charge state of droplet
   to be considered - 8 times the average size of a log-normal distribution
   LogNor = np.zeros(fields)
 # determine intensity for each charge state from log-normal

   for i in range (1,fields):
      x=SizePerCharge/AverageNeutral*i
# the x value is 1 for the average size and all multiples of the selected mass per charge are
   considered up to the max charge state

      LogNor[i]=m.exp(-(m.log(x)*m.log(x)/2.0))/x
# log-normal distribution with sigma = 1 and μ = 0

   LogNor=LogNor/np.amax(LogNor)
   clus = np.zeros(max_c)
# array for charges in the droplet that grow to singly charged dopant clusters

   last = np.zeros(max_c)
# array for the last charge state in the case that all other ions are ejected in the pickup cell -
   this one has more He available to grow

   SizeCritC = np.zeros(fields)
   for c in range(1, fields):
      SizeCritC[c] = 91.316 * m.pow((c + 1.544) / 0.026, 3.0 / 2.0)
# 4/3*pi*21.8*r^3 and r^2(z)=(z+0.544)/0.026 - values taken from Laimer et al. PRL 2019 and
   density of liquid He

   for c in range(1,fields):
      nHe=SizePerCharge*c
      if nHe>SizeCritC[c]:
         lamda_c = 0.000769794 * m.pow(nHe,2.0 / 3.0)
# expectation value for charging a droplet with nHe atoms with a 500μA electron current,
   1mm^2 with a speed of 200 m/s (has to be adjusted for other ion source settings)

         prob_c=Poisson(c,lamda_c)*LogNor[c]
 # probability for obtaining a droplet with a specific mass per charge value (Poisson electron
   ionization and log-normal neutral size distribution)
```

```
            for k in range(int(runs*prob_c)):
                nHe=SizePerCharge*c*(random.random()*0.1+0.95)
# adds ±5% scatter to droplet size to account for the resolving power of the quadrupole
  bender

                sigma=sigma0*m.pow(float(c/fields),2.0/3.0)
# cross section scales with size of the droplets and z charges increases the value by z^(2/3)

                f = np.zeros(c)
                out=0
# out is the number of charges that were removed from the droplet via Coulomb repulsion

                for i in range(PU_Dens):                    # vapor pressure in PU cell
                    nHe=nHe-HeLoss*c                        # He loss due to collisions with He
                    if (sigma>random.random()) and (nHe>evap):
# with decreasing sigma, the chance for pickup goes down and we need at least enough He to
  evaporate the 1600 He atoms times the released energy (evap)

                        hit=int(random.random()*(c-out))
                        f[hit]=f[hit]+1
                        count=count+1
                        sigma=sigma*m.pow((nHe-evap)/nHe,2.0/3.0)
# reduction of pu cross section due to shrinking of droplet

                        nHe=nHe-evap
                        if (nHe<SizeCritC[c-out]) and (out<c):
# check if size is below critical value for Coulomb repulsion

                            maxcs=0
                            for j in range(1,c-out):
# switches the largest dopant cluster with the one at the last available position in the array
  and increases out
                                if f[j]>f[maxcs]:
                                    maxcs=j
                            out = out + 1
                            if maxcs<c-out:
                                help=f[c-out]
                                f[c-out]=f[maxcs]
                                f[maxcs]=help
                if out == c:
# only a single charge is in the droplet and there is no critical size for Coulomb repulsion - in
  the best case, this cluster has 10^5 He atoms to grow

                    for i in range(1,c):
                        clus[int(f[i])] = clus[int(f[i])] + 1
                    last[int(f[0])] = last[int(f[0])] + 1
                else:
                    for i in range(c):
                        clus[int(f[i])] = clus[int(f[i])] + 1
    norm=np.amax(clus)
    clus = clus/norm
```

```
    last = last/norm
    Deviation = 0.0
    for i in range(min(len(clus),len(experiment))):
        Deviation=Deviation+(clus[i]-experiment[i])*(clus[i]-experiment[i])
    if PlotGraph:
        print(count, Deviation)
        plt.plot(clus+last)
# plots the simulated cluster size distribution (orange line)

        plt.plot(last)
# plots contribution of last charge in droplet

        f = open('test.txt','w')
# writes the simulated cluster size distribution to a file

        for i in range(len(clus)):
            line=str(i+1) + '\t' + str(clus[i]+last[i]) + '\t' + str(clus[i]) + '\t' + str(last[i]) + '\n'
            f.write(line)
        f.close()
    return(Deviation)

# load of experimental data
table = np.genfromtxt('Au13.dat', delimiter='\t')
experiment=np.zeros(len(table))
for i in range(len(table)):
    experiment[i]=table[i,1]
experiment=experiment/np.amax(experiment)
plt.plot(experiment)                          # plots the experimental cluster size
    distribution (blue line)
LossPerPU = 4020                  #He loss per pick up
Density=1900                      #Density of dopant atoms
max_c=500
AverageNeutralSize = 9125000          #Average size of neutral He droplet
HePerCharge = 1.45e5              #He atoms per charge
ChiSquare=CalcDist(AverageNeutralSize, HePerCharge, LossPerPU, 7000.0, Density, 80.0, 1.0,
    True)
plt.xlim(0,100)
plt.show()
    # print(clus)
```
