# Supplementary material for: Efficient Formation of Size-Selected Clusters upon Pickup of Dopants into Multiply Charged Helium Droplets
Source: Int J Mol Sci. 2022 Mar 25;23(7):3613. doi: 10.3390/ijms23073613 (PMC8998201; doi:10.3390/ijms23073613)
Supplement: Supplementary file 1 [file ijms-23-03613-s001.zip › Table S1-The variables used for simulations of the cluster size distributions and corresponding parameters estimated from the experiment.pdf]

Table S1: The variables used for simulations of the cluster size distributions and corresponding parameters estimated from the experiment.

element	Au	Au	Na	Na
n,max	33	13	3	34
LossPerPU	4020	8020	9900	1800
Density	1900	560	540	601
AverageNeutralSize	9.125×10^5	7.12×10^5	3.125×10^5	2.75×10^5
HePerCharge	1.45×10^5	1.22×10^5	5.4×10^4	1.06×10^5
Estimated amount of He atoms per charge	3.39×10^5	1.14×10^5	4.57×10^5	5.64×10^5
Estimated droplet size	1.8×10^6	6.25×10^5	2×10^6	2×10^6

Large values for He loss per pickup (LossPerPU) found for Na with n, max=3 (9900, instead of approx. 1000) that are way above the binding energy can be interpreted as a result of the heliophobic nature of sodium. If the droplet size (AverageNaturalSize) is only 5.4×10^5, the droplets cannot be highly charged. Most probably the droplets are singly charged and the charge is located inside the droplet. Therefore, many Na atoms may hit the droplet and will desorb before reaching the charge center.
